# Protocol for Project FACT: a randomised controlled trial on the effect of a walking program and vitamin B supplementation on the rate of cognitive decline and psychosocial wellbeing in older adults with mild cognitive impairment [ISRCTN19227688]

**DOI:** 10.1186/1471-2318-5-18

**Published:** 2005-12-23

**Authors:** Jannique GZ van Uffelen, Marijke Hopman-Rock, Marijke JM Chin A Paw, Willem van Mechelen

**Affiliations:** 1 Body@Work, Research Center Physical Activity, Work and Health, TNO-VU University Medical Center, Amsterdam, The Netherlands; 2 Department of Public and Occupational Health, EMGO Institute/VU University Medical Center, Amsterdam, The Netherlands; 3 Dep. of Physical Activity and Health, TNO Quality of Life, Leiden, The Netherlands

## Abstract

**Background:**

the prevalence of individuals with cognitive decline is increasing since the number of elderly adults is growing considerably. The literature provides promising results on the beneficial effect of exercise and vitamin supplementation on cognitive function both in cognitively healthy as well as in the demented elderly.

**Methods/Design:**

the design is a two-by-two factorial randomised controlled trial. The study population consists of independently living elderly, between 70 and 80 years old, with mild cognitive impairment (MCI). In the RCT the effect of two interventions, a walking program and vitamin supplementation, is examined. The walking program (WP) is a group-based program aimed at improving cardiovascular endurance; frequency two lessons a week; lesson duration one hour; program duration one year. Non-walking groups receive a placebo activity program (PAP) (i.e. low intensive non-aerobic group exercises, like stretching) with the same frequency, lesson and program duration. Vitamin supplementation consists of a single daily vitamin supplement containing 50 mg B6, 5 mg folic acid and 0,4 mg B12 for one year. Subjects not receiving vitamin supplements are daily taking an identically looking placebo pill, also for a year. Participants are randomised to four groups 1) WP and vitamin supplements; 2) WP and placebo supplements; 3) PAP and vitamin supplements; 4) PAP and placebo supplements. Primary outcome measures are measures of cognitive function. Secondary outcomes include psychosocial wellbeing, physical activity, cardiovascular endurance and blood vitamin levels.

**Discussion:**

no large intervention study has been conducted yet on the effect of physical activity and vitamin supplementation in a population-based sample of adults with MCI. The objective of the present article is to describe the design of a randomised controlled trial examining the effect of a walking program and vitamin B supplementation on the rate of cognitive decline in older adults with MCI.

## Background

Because cognitive function decreases with age and the number of elderly is increasing worldwide, the number of older adults with cognitive dysfunction is also increasing. Since no cure for dementia is available yet, this process will put a considerable burden on the healthcare system and on society in general. Therefore, for both individuals and society it is necessary to develop strategies for maintaining physical, mental and cognitive wellbeing of the aging population.

In this manuscript the design is described of a randomised controlled trial on the effect of a moderate intensive walking program and vitamin B-supplementation in an older population with mild cognitive impairment (MCI) recruited from the general population.

### Mild cognitive impairment

MCI refers to a stage in which persons experience memory loss to a greater extent than one would expect for age, but do not yet meet currently accepted criteria for clinically probable dementia or Alzheimer Disease (AD) [1]. Prevalence of MCI in adults with a mean age of 75 years is three to four percent [2,3]. Compared to elderly with normal cognitive function, adults with MCI have an increased risk to develop dementia or AD [1,4,5]. Progression rates to dementia and AD for adults with MCI vary from 6 % to 25 % per year, depending on the criteria for MCI [6]. The rate of progression to AD in adults with MCI according to the criteria of Petersen et al. is 12%, compared with a rate of 1–2% per year in control subjects [4].

As the stage of MCI may be the optimum stage at which to intervene with preventive therapies [7,8], new treatments to prevent development of AD are targeting elderly with MCI [7].

### Physical activity

The hypothesis of physical activity positively influencing cognition is supported by observational studies that found higher levels of physical activity were associated with a reduced risk of cognitive decline and dementia in healthy elderly [9-13].

Positive effects of exercise programs on cognitive function have been found also in various trials among cognitively healthy elderly [14-17]. Programs lasted from two months [14] to one year [16], frequency and intensity differed per program. Improvements in memory [14], immediate recall [16,17] and tasks requiring executive control processes [15] have been reported.

Also a number of reviews and a meta-analysis have been conducted on the effects of physical exercise on cognition in healthy elderly [18-20]. Exercise seems to have a positive effect on cognitive functioning, but findings in the individual studies are contradictory and the effect size can be considered small. However, two recently published meta-analyses reported moderate effect size values, around 0.5, from group based aerobic fitness training on cognitive performance in healthy elderly [21] and from various types of exercise programs in elderly with dementia [22].

Besides an effect on cognitive function, Biddle and Faulkner [23] concluded in their review that clear beneficial effects from physical activity are evident for psychosocial wellbeing in older adults.

Possible pathways for the effect of physical activity on cognitive function are increased blood flow to the brain, improved vascularisation, improved neurogenesis, increased neurotransmitter availability and better neural efficiency[24,25].

### Vitamin B supplementation

Cognitive function may benefit as well from supplementation with folic acid, vitamin B12 and vitamin B6 (FA/B12/B6 respectively). Metabolic FA/B12/B6 deficiencies are relatively common in older adults [26,27], even in the presence of normal serum vitamin levels [28]. Low levels of FA/B12/B6 seem to be associated with poorer cognitive function [29-31]. Besides a reverse effect on cognitive function, FA/B12/B6 deficiencies result in elevated levels of the amino acid homocysteine [32], as these vitamins are linked to the metabolism of homocysteine.

An elevated homocysteine level has been found to be a strong independent risk factor for the development of dementia and AD as well [33]. Negative associations have been found between increased homocysteine levels and global cognitive performance [34-36], memory [37,38], psychomotor speed [38,39] and spatial copying skills [29], even in the generally normal accepted range of homocysteine (25^th^–75^th ^percentile (7.6–11.3 μmol/L)) [38]. Above a threshold of approximately 14 μmol/L significantly lower cognitive performances were observed as well [39-41].

Supplementation with vitamin B reduces the homocysteine level [42,43]. Supplementation with folic acid after standardisation for pre-treatment blood concentrations of homocysteine and folic acid resulted in an approximate decrease of homocysteine level of 25 percent. Vitamin B12 produced an additional 7 percent reduction [44].

Few experimental studies have been conducted on the effect of B-vitamin supplementation in enhancing cognitive performance in older adults. Bryan and Calvaresi (2002) [45] found a significant effect on memory performance after 35 days of (FA/B12/B6) supplementation in healthy middle aged and older women. Fioravanti et al. [46] concluded in a double blind controlled trial that 15 mg folic acid daily for 60 days given to older adults with global impairment in all components of memory functioning (i.e. a more seriously decline than MCI), appeared to improve their memory. Vitamin B12 injections of different doses improved memory in elderly with B12 deficiency and without cognitive impairment in a single blind controlled trial [47]. Finally, cognitive performance in elderly with elevated plasma homocysteine and a diagnosis of mild to moderate dementia improved after combined supplementation of 5 mg folic acid and 1 mg vitamin B12 daily for two months [48]. Unfortunately most of these trials included few subjects and, therefore, results have to be interpreted with care.

Calvaresi and Bryan (2001) hypothesised that FA/B12/B6 may affect cognitive performance via two interrelated ways: a direct and possibly acute influence via hypomethylation and a longer term influence on homocysteine levels resulting in structural vascular changes in the brain [49].

No clinical trials are yet available that have examined the effect of physical activity and vitamin supplementation in a population based sample of adults with MCI. Therefore, the main objective of project FACT (Folate physical Activity Cognition Trial) is to examine the effect of a walking program and vitamin B supplementation on the rate of cognitive decline in adults aged 70 to 80 years with mild cognitive impairment in a randomised controlled trial. Also effects of both interventions on psychosocial wellbeing, habitual physical activity and cardiovascular endurance will be examined.

## Methods/Design

### Study design

**Figure 1:**
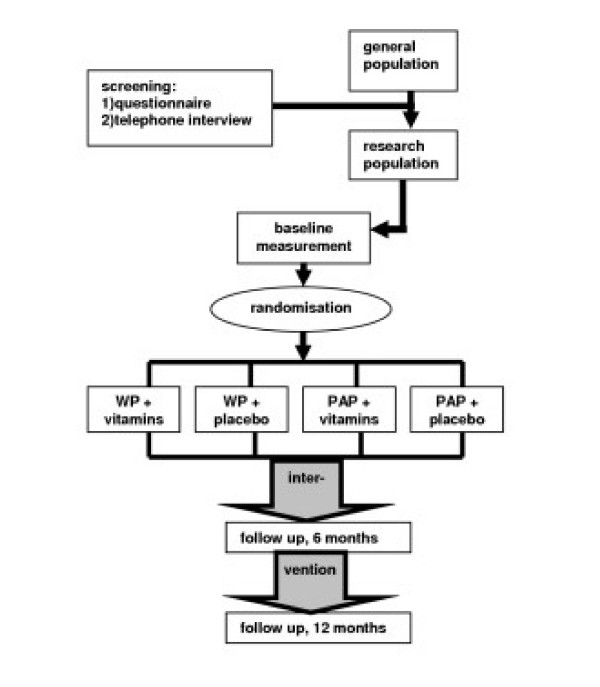
Design of the RCT (WP = walking program, PAP = placebo activity program).

The study is designed as a randomised, placebo controlled intervention trial, based on a two-by-two factorial design. The design is presented in figure 1. It is assumed that the effect of both interventions is independent. The study protocol was approved by the VU University Medical Center medical ethics committee.

### Setting

This study is carried out in Alkmaar, a medium sized city in The Netherlands, with approximately 100,000 inhabitants.

### Study population

This study is targeted at a population based sample meeting the criteria for mild cognitive impairment: i.e. memory complaint, memory impairment, normal general cognitive function, normal activities of daily living and not demented [4]. In the recruitment procedure the criteria for MCI are checked in both a questionnaire and a telephone interview. Other inclusion criteria for participating in the trial are checked for only in the questionnaire (table 1).

**Table 1:** Inclusion criteria

Criteria for MCI (1–4) and other inclusion criteria for the RCT (5–11)

1. self reported memory complaints (answer yes to question 'do you have memory complaints', or at least twice sometimes at cognition scale of Strawbridge)
2. no report of disability in activities of daily living on GARS-scale, except on the item 'taking care of feet and toe nails'
3. objective memory impairment; 10 wlt delayed recall ≤5 + percentage savings ≤ 100
4. normal general cognitive functioning/absence of dementia; TICS ≥ 19
5. being able to moderate intensive physical activity without making use of walking devices, e.g. rollator, of walking frame
6. not using vitamin supplements/vitamin injections/drinks with dose of vitamin B6, B11 or B12 comparable to vitamin supplement given in intervention
7. not suffering from epilepsy, multiple sclerosis, Parkinson's disease, kidney disorder requiring haemodialysis, psychiatric impairment
8. not suffering from depression as measured by the Geriatric Depression Scale (cut off ≤ 5)
9. not using medication for rheumatoid arthritis or psoriasis interfering with vitamin supplement
10. no alcohol abuse (men < 21 consumptions a week, women < 15 consumptions a week)
11. not currently living in or on a waiting list for a nursing home

#### Questionnaire

To recruit participants a questionnaire is sent to all independently living elderly in Alkmaar, with an age between 70 and 80 years old. Their addresses are provided by the register of population of the municipality of Alkmaar. Primary aim of the questionnaire is to check two criteria for MCI, memory complaints and normal activities of daily living. Memory complaints are assessed by the question 'do you have memory complaints' and the cognition scale of Strawbridge [50]. Activities of daily living (ADL) are assessed using the Groningen Activity Restriction Scale (GARS) [51]. Secondary aims are to collect demographic variables and to check the other inclusion criteria for trial participation. Together with filling in the questionnaire, participants are requested to give informed consent.

#### Telephone interview

Respondents fulfilling the inclusion criteria are being phoned for a telephone interview for cognitive status. In this telephone interview a brief measure of general cognitive function (TICS) [52] and a modified version of the ten word learning test [53] are administered. Adults with a score of 20 or more on the TICS, corresponding to normal cognitive function, and with a performance on the delayed recall score on the ten word learning test of less than five are considered as having mild cognitive impairment. A delayed recall score of five or less corresponds with one standard deviation below normal performance [54]. Participants fulfilling these criteria receive an invitation letter for a baseline measurement during a personal interview.

### Sample size

The aim is to enrol 170 participants. A power analysis has been executed on the auditory verbal learning test. To be able to detect a difference of 5 points on direct recall (see 2.9.1) 34 participants per group are required and thus 136 participants in total. These numbers are based on a power of 80% and a significance level of 0.05. We expect a drop-out rate of 25 percent, based on experiences with comparable research. Therefore, 170 participants will be recruited.

### Randomization

To ensure an equal distribution of physically inactive and active subjects in each group, participants are classified as active or inactive on the basis of their activity level as measured using the LASA physical activity questionnaire [55]. Adults with an activity level exceeding the median level of the whole group are classified as active and adults below this level are classified as inactive. Active and inactive subjects are allocated separately and randomly to one of the four intervention groups using the option 'random sample of cases' in the statistical computer program SPSS.

### Blinding

The study is conducted double blind. The key of coding for FA/B12/B6 supplementation is only known to the manufacturer of the supplements, who will decode the key after data-analysis. All outcome measures on cognition and wellbeing are assessed by independent examiners unaware of group allocation.

### Co-interventions and compliance

During the intervention period co-interventions are discouraged by asking adults not to start an exercise program or vitamin supplementation while being a participant. Both vitamin supplementation and physical activity level will be asked for at baseline and after 6 and 12 months.

Compliance with the walking program is assessed as the percentage of attended lessons. Compliance with the vitamin supplementation is verified by pill counts and determining blood vitamin levels.

### Intervention

#### Walking program and placebo activity program

##### Walking program (WP)

The walking program is based on 'Sportive Walking', an existing aerobic exercise program [56]. Each lesson consists of a warming-up, moderate intensity walking exercises and a cooling-down. Lessons for project FACT are developed by two certified walking instructors and recorded in a manual. The duration and intensity of the WP are increased gradually during the program by increasing the total walking time and distance. The WP is developed in such a way that all subjects can perform the walking exercises at their own level, but still walk in a group. This is for example established by using walking routes with the same beginning and end.

Lessons are being given outdoors in municipal parks in Alkmaar. Only when it is slippery due to snow or freezing rain the lessons will be cancelled.

##### Placebo activity program (PAP)

The PAP is developed by four experienced exercise instructors. The program consists of an introduction, low intensity non-aerobic group exercises such as light range of motion movements and stretching, and a closing. Lessons are divided into five themes: relaxation, activities of daily living, balance, flexibility, posture, and a combination of all. For each theme three lessons are developed and the entire series of 18 lessons is repeated during the intervention period. The program takes place in a community center.

Both programs are group-based and last one year, the frequency is twice a week and lesson duration is 60 minutes. The intensity is checked by heart rate monitors (Polar, Vantage NV) and Borg scales in a sub sample of the population during one lesson at baseline and after twelve months and during two lessons at six months follow up.

To provide an intervention class in or near the subjects' own neighbourhood, eight classes for the walking program and eight classes for the placebo activity program are started in four districts. These classes are organized especially for the study and only study participants are able to join. All exercise classes are supervised by qualified instructors. In total four trained walking instructors for the walking program and four exercise instructors for the placebo activity program are hired for the study. The number of participants in a group is 19 at the most.

#### Folic acid/vitamin B12/vitamin B6 supplementation

Subjects in the intervention group are asked to take daily one pill containing 5 mg folic acid, 0.4 mg vitamin B12 and 50 mg B6 during a year. Subjects who do not get FA/B12/B6 supplementation receive an identically looking placebo pill. The pills are packed in blister packs containing seven pills that are labelled for each day of the week.

### Measurements

All outcome measures are collected at baseline and after 6 and 12 months. The primary outcome measures are measures of cognitive function. Secondary measures are measures of psychosocial health, habitual physical activity and cardiovascular endurance. Also physical measures are performed. Most cognitive and psychosocial data are gathered during a standardized interview. These face-to-face interviews are conducted in the Medical Center Alkmaar and last a maximum of 90 minutes, including a short break.

#### Primary outcome measures

Five interviewer administered cognitive outcome measures are chosen to assess different aspects of cognitive functioning. Only the informant questionnaire is self administered.

##### Mini Mental State Examination (MMSE)

general cognitive function is measured with the MMSE. The MMSE consists of 11 questions concerning orientation, registration, attention and calculation, recall and language. The maximum score is 30 and a score below 24 is considered abnormal for dementia screening [57].

##### Auditory Verbal Learning Test (AVLT)

a Dutch version of the AVLT is used. This is a measure of memory in which direct and delayed recall are assessed. During the test a list of 15 monosyllabic words is read aloud by the examiner for 5 times. After each trial the subject is asked to repeat the words he or she remembers. After fifteen minutes with other questions, delayed recall is assessed by asking the participant which words he or she still remembers [58]. At baseline and after 12 months the same version of the test is administered and after 6 months a parallel version with 15 different words is administered.

##### Letter fluency test (LFT)

this is a measure of expressive language. If language is intact, the LFT is also a measure of executive functioning. The subject is given a letter and is asked to name words beginning with the particular letter in one minute. In one administration of the test three letters are given. At the 6 and 12 months follow up measurements parallel versions with different letters are administered [59].

##### Digit symbol substitution test (DSST)

This is a measure of attention, perceptual speed, motor speed, visual scanning and memory. The subject is given a piece of paper with nine symbols corresponding with nine digits. Next on this piece of paper are three rows of digits with empty spaces below them. The subject is asked to fill in as many corresponding symbols as possible in 90 seconds [60].

##### Abridged Stroop colour word test (SCWT-A)

this is a measure of complex processing. The SCWT-A consist of three tasks; 1) word reading, 8 rows of 5 written colours; 2) colour naming, naming the colours of 8 rows of 5 red, green, blue or yellow coloured rectangles; 3) combination task, the words *red, green, blue *or *yellow *have been printed in a different colour of ink, the subject is asked to name the colour of the ink [61].

##### Informant questionnaire on cognitive decline (IQ-code)

in this questionnaire a significant other of the participant is asked to answer 16 questions about changes in the participant's cognitive function during the last ten years [62,63].

##### Secondary measures

Three interviewer administered questionnaires are used to complete the picture of psychosocial health. Only the geriatric depression scale is self administered.

##### psychosocial wellbeing

##### Short Form 12 (SF-12)

The SF-12 is a measure of health status consisting of twelve items measuring eight concepts of both physical and mental health. The physical and mental component summary scales are scored using norm-based methods [64].

##### Dementia Quality of Life (D-QoL)

The D-QoL is a 29 item measure especially developed for elderly with cognitive decline and dementia. Five domains are measured: self esteem, positive affect/humour, negative affect, feelings of belonging and sense of aesthetics. The response scale is a five point scale with higher score indicating better quality of life [65].

##### Euro Quality of Life (Euro-QoL)

The Euro-QoL questionnaire is a standardized measure for general health status measuring five dimensions: mobility, self care, usual activities, pain/discomfort and anxiety/depression. The participant is asked to choose from three answer levels for each dimension. By composing a five digit number consisting of the three answer levels on the five dimensions participants can be classified into one of 243 defined health states [66].

##### Geriatric Depression Scale (GDS)

The GDS is a self administered depression scale for the elderly. The short version is used in which participants are asked to report how they felt the last week by answering 15 yes/no questions. The maximum score is 15 and a score over five points is suggestive of depression [67].

##### physical activity and cardiovascular endurance

##### Physical activity level

During the face-to-face interview the LASA physical activity questionnaire [55] is administered to determine the physical activity level of the participants. Participants are interviewed about their physical activities during the last two weeks by asking questions about the frequency and duration of different activities (e.g. housekeeping, sports activities, cycling, gardening). These answers are converted to an overall physical activity score expressed in minutes of physical activity per day.

##### Accelerometer

A random sample of subjects of each of the four intervention groups is asked to wear an accelerometer (ActiGraph, activity monitor) for three days. Data will be used to compare the level of activity in participants in the walking program and in the placebo activity group.

##### Cardiovascular endurance

this is assessed in a sports hall using the walking test of the Groningen Fitness test for the elderly [68]. This is a sub-maximal test for aerobic endurance in which adults walk distances of 16.6 meters between pylons in a large rectangle of 16.6 by 8.3 meters. For every walked distance a score of one point is given. To increase their score, adults have to be within a three meter distance of the next pylon before a signal sounds. A double signal sounds when walking speed is increased. Walking speed is increased every three minutes with one kilometre starting with a walking speed of four kilometres per hour to a maximum walking speed of seven kilometres per hour. If they fail twice to reach the next pylon in time, the test is finished for that particular participant. The maximum score of 66 points corresponds to a total walking distance of 1.1 kilometres.

#### Physical measures

##### Anthropometric measurements

During the break in the face-to-face interview body height and body weight are measured for calculating body mass index (kg/m^2^).

##### Blood pressure

Blood pressure is measured electronically (Omron M5-1) after five minutes of rest during the face-to-face interview. Participants with a diastolic blood pressure exceeding 95 and a systolic blood pressure exceeding 160 are offered to have their blood pressure measured again by a geriatrician. If hypertension is diagnosed, the participant will be treated.

##### Blood vitamin levels

non-fasting blood samples are taken at the laboratory of the Medical Center Alkmaar to determine blood vitamin levels.

Plasma concentrations of homocysteine, serum folate levels and vitamin B12 levels in serum are determined by a competitive immunoassay using direct chemiluminescent technology. (ADVIA CENTAUR, Bayer Corporation, Tarrytown, USA).

For determination of red cell folate hemolysates are prepared out of EDTA plasma and ascorbic acid. Folate in erythrocytes is determined in the hemolysate by a competitive immunoassay using direct chemiluminescent technology (ADVIA CENTAUR, Bayer Corporation, Tarrytown, USA). According to the manufactures instructions folate in erythrocytes is calculated using the following algorithm: folate in hemolysate × 21 minus folate in serum × ((100 – hematocrit)/(hematocrit * 100)).

Vitamin B6 in plasma is determined by high performance liquid chromatography with fluorescence detection using Chrompack Lichrosorb RP-18 columns (Varian Inc., Palo Alto, USA) and the Jasco HPLC system (Jasco Benelux, Maarssen, The Netherlands).

### Statistical analyses

Analyses will focus upon estimating the effect of both interventions on four domains: 1) cognitive functioning; 2) psychosocial wellbeing; 3) habitual physical activity and cardiovascular endurance; 4) blood vitamin levels and homocysteine. The effect of physical activity and FA/B12/B6 supplementation will be examined independently from each other as we do not expect an interaction between these interventions.

Before analyses are performed there will be a check-up on the comparability at baseline of the intervention groups. If necessary, analyses will be adjusted for baseline differences. Subsequently, the data set will be analysed according to the intention to treat and according to the intention per protocol principle.

The difference between the walking program and the placebo activity program, and between the verum and placebo supplements will be assessed using linear regression. The dependent variables will be values after 6 and 12 months in the four before mentioned domains. Both interventions will be independent variables. Besides, regression analyses will be adjusted for baseline values and possibly confounding covariates such as gender and age. Also effect modification by sex will be investigated using interaction terms.

## Discussion

The literature provides promising results on the beneficial effect of exercise and folic acid/vitamin B12/vitamin B6 supplementation on cognitive function and wellbeing both in cognitive healthy as well as in the demented elderly. However, to our knowledge, no large intervention study has been conducted yet on the effect of these interventions on cognitive decline in subjects with MCI. In the present study, the effects on cognitive function and psychosocial health will be examined in a population sample of community dwelling elderly adults aged 70 to 80 years. A factorial design is used and it is assumed that the interventions have an independent effect. We hypothesize that both exercise and FA/B12/B6 supplementation beneficially influence cognitive function and psychosocial health in this particular group of elderly.

The results of this trial will provide clinicians in the field of aging with more knowledge about treatment of older persons with cognitive decline. If proven effective, exercise and vitamin supplementation are an additional intervention method for this target group.

Implementation of the walking program in the Netherlands is relatively easy, since infrastructure for group based exercise programs for the elderly already exists.

Vitamin supplementation is easy to implement as well. The most difficult aspect might be long term compliance in case of severe cognitive decline. However, compliance can be sustained by providing methods to remember taking the vitamin supplements.

An additional important advantage of both interventions is that involvement of the clinician is limited to prescribing exercise and vitamin supplementation and possibly to evaluate compliance at future medical check-ups.

## Abbreviations

AVLT: auditory verbal learning test

D-QoL: dementia quality of life

DSST: digit symbol substitution test

Euro-QoL: European quality of life

FA/B12/B6: folic acid, vitamin B12, vitamin B6

GARS: Groningen activity restriction scale

GDS: geriatric depression scale

IQ-code: informant questionnaire for cognitive function

LFT: letter fluency test

MCI: mild cognitive impairment

MMSE: mini mental state examination

PAP: placebo activity program

SCWT-A: stroop color word test abridged

SF-12: short form 12

TICS: telephone interview for cognitive status

WP: walking program

## Competing interests

The author(s) declare that they have no competing interests.

## Author's contributions

MC was involved in developing the basic idea for the study. JvU, MH, MC and WvM were involved in further developing the idea and the protocol for carrying out the study. JvU is responsible for the data collection and she drafted the manuscript. All authors contributed to the final manuscript by reading and correcting draft versions.

## Pre-publication history

The pre-publication history for this paper can be accessed here:


